# The Gray Mold Spore Detection of Cucumber Based on Microscopic Image and Deep Learning

**DOI:** 10.34133/plantphenomics.0011

**Published:** 2023-01-10

**Authors:** Kaiyu Li, Xinyi Zhu, Chen Qiao, Lingxian Zhang, Wei Gao, Yong Wang

**Affiliations:** ^1^ China Agricultural University, Beijing, 100083, China.; ^2^ Key Laboratory of Agricultural Informationization Standardization, Ministry of Agriculture and Rural Affairs, Beijing, 100083, China.; ^3^ Tianjin Academy of Agricultural Sciences, Institute of Plant Protection, Tianjin, 300384, China.

## Abstract

Rapid and accurate detection of pathogen spores is an important step to achieve early diagnosis of diseases in precision agriculture. Traditional detection methods are time-consuming, laborious, and subjective, and image processing methods mainly rely on manually designed features that are difficult to cope with pathogen spore detection in complex scenes. Therefore, an MG-YOLO detection algorithm (**M**ulti-head self-attention and **G**host-optimized **YOLO**) is proposed to detect gray mold spores rapidly. Firstly, Multi-head self-attention is introduced in the backbone to capture the global information of the pathogen spores. Secondly, we combine weighted Bidirectional Feature Pyramid Network (BiFPN) to fuse multiscale features of different layers. Then, a lightweight network is used to construct GhostCSP to optimize the neck part. Cucumber gray mold spores are used as the study object. The experimental results show that the improved MG-YOLO model achieves an accuracy of 0.983 for detecting gray mold spores and takes 0.009 s per image, which is significantly better than the state-of-the-art model. The visualization of the detection results shows that MG-YOLO effectively solves the detection of spores in blurred, small targets, multimorphology, and high-density scenes. Meanwhile, compared with the YOLOv5 model, the detection accuracy of the improved model is improved by 6.8%. It can meet the demand for high-precision detection of spores and provides a novel method to enhance the objectivity of pathogen spore detection.

## Introduction

China's vegetable industry is developing rapidly, and the total area under greenhouse cultivation now ranks first in the world [1,2]. Cucumber is one of the top 10 vegetables in the world and is famous for its high food, nutritional, and medicinal values. However, the greenhouse temperature and humidity during cucumber cultivation are favorable for the reproduction and spread of fungal diseases (cucumber gray mold, downy mildew, etc.). In recent years, the area under cultivation has expanded further and the incidence of disease has then become more serious. In severe cases, this can lead to 20% to 50% yield loss or even no harvest [3,4]. If the fungal spores that cause vegetable diseases can be detected in the early stage of disease, and certain preventive measures can be taken, the effect will be better. In addition, statistical information on fungal spores during disease control drug development can reveal the drug resistance degree and activity of spores [5], thus providing strong technical support for research on novel biological or chemical drugs. Therefore, accurate and real-time detection of fungal spores from greenhouse crops is necessary to study fungal and crop diseases.

Traditional spore detection methods include microscopic observation, molecular biology, and microscopic image processing-based methods. The traditional microscopic observation method has the disadvantages of high workload, low efficiency, and reduced accuracy with longer working time. The molecular biology method of DNA sequence identification for quantitative detection has the advantages of objectivity, precision, and high throughput [6,7]. Still, it requires specialized personnel and is time-consuming, laborious, and expensive [8].

The microscopic image processing method is based on image processing and machine learning methods to detect spores. Researchers first preprocess the spore microscopic images with grayscale, median filtering and then utilize threshold analysis, edge detection, region growth, and grayscale co-occurrence matrix to extract features such as color, shape, and texture to detect spores [9,10]. Tahir et al. [11] first extracted patches of size 78 × 78 × 3 from the spores’ images, then extracted histogram of oriented gradient features of patches and manual features such as color, size, and shape separately, and finally identified spores’ patches by a support vector machine. This method achieved an average precision (AP) of 88.12% when the spore’s density was small (number ≤ 50). However, as the spore density increased, the algorithm faced difficulty and reached its limits. Lei et al. [12] chose the shape factor of target contour to identify touching urediniospores. Yang et al. [13] selected 7 features such as ellipticity, complexity, and entropy to construct the decision tree model. The detection accuracy is as high as 94%. Wang et al. [1] extracted 90 features containing fungal spore color, shape, and texture and proposed a multifeature fusion method based on a support vector machine. The above study can detect nonadherent spores well and has the characteristics of fast and efficient. These methods depend on the important feature type of the object, so they are not very portable and general and are not robust and accurate for spore detection in complex multiadhesion cases.

Deep convolutional neural network (DCNN)-based object detection dramatically improves the detection accuracy of vision. It obtains high-dimensional features after multilayer convolutional processing with strong robustness and antinoise, antidefect ability [14]. The detection methods of DCNN include 2-stage networks and 1-stage networks. The representative 2-stage networks are Region-CNN (RCNN) [15], Fast RCNN [16], and Faster RCNN [17,18]. Zhang et al. [18] proposed FSNet, a model for the detection of fungal spores during grain storage, which incorporates deep, intermediate, and shallow features of feature extraction networks and combines clustering losses to reduce missed detections of aggregated spores with an AP higher than 0.9. It has a slow detection speed and a small density of fungal data. Representative single-stage networks are SSD [19], RetinaNet [20], and YOLO [21] series models. These networks treat object detection as a regression problem, with high real-time performance. Recently, the 1-stage YOLO series detection networks have been widely used in various agricultural scenarios, including disease identification [22], fruit detection [14,23], pest detection [24], etc. Among the YOLO series, the structure of YOLOv5 is further optimized to make object detection fast and accurate and is favored by researchers [23,25]. YOLOv5 adds Mosaic enhancement and adaptive anchor box at the data input side. The feature extraction network based on CSPDarknet53 can reduce memory loss within a certain range [26]. The processing output part uses Feature Pyramid Networks (FPN) [27] and Path Aggregation Network (PANet) structure [28], which can speed up the information flow between the layers. Its potential in fungal spore detection has not been verified yet.

In microscopic images, the polymorphic, small-target, and heavily obscured nature of spores makes fungal spore detection challenging. In the early stage, we used the original YOLO algorithm for detection, and the results were unsatisfactory. In this paper, we propose an improved YOLOv5 (**M**ulti-head self-attention and **G**host-optimized **YOLO** [MG-YOLO]) spore detection method to solve these problems and improve the accuracy of spore detection in high-density scenarios. The main objectives include the following:

1. We propose a backbone with the introduction of the Multi-head self-attention (MHSA). The MHSA dramatically improves the ability of the network to model remote dependencies to enhance the model's accuracy in detecting relatively deep spores.

2. Optimizing PANet using weighted Bidirectional Feature Pyramid Network (BiFPN) enhances feature convergence at no additional cost, reducing missed fungal spore detection. Ghost model to optimize the neck to decrease the number of parameters in the model.

3. A novel detection method called MG-YOLO is proposed for detecting fungal spores; extensive experiments have shown that MG-YOLO has good trade-offs in terms of detection efficiency and accuracy in multiple scenarios and densities.

The remainder of this paper is organized as follows. The “Dataset generation” and “The proposed MG-YOLO architecture for spore detection” sections describe the gray mold spore dataset and the methods used to detect it in this study. The “Results and Discussion” section shows the results and discussion of the proposed methods. Finally, we give conclusions and future work in the “Conclusion” section.

## Materials and Methods

### Dataset generation

#### Image acquisition

The experiment test strain was HGHM01, which belonged to the asexual type of *Botrytis cinerea*, obtained from the Institute of Plant Protection, Tianjin Academy of Agricultural Sciences. The test strain HGHM01, which was stored at 4 °C, was purified on potato dextrose agar medium and placed in a constant temperature incubator at 28 °C in the dark for 5 d. After 5 d, a layer of gray-brown mold grew on the surface of the medium. Then we added 10 ml of sterile water to the potato dextrose agar petri dishes and scraped off the mycelial spores with a scraping stick. Finally, the mycelium was removed by filtering with double gauze. Then a concentration of 1 × 10^7^ spores/spore ml of suspension was observed and configured by a blood counting chamber, finally diluted with sterile water to a spore concentration of 1 × 10^6^, 1 × 10^5^, and 1 × 10^4^ spores/ml spore suspension for backup.

**Fig. 1.**
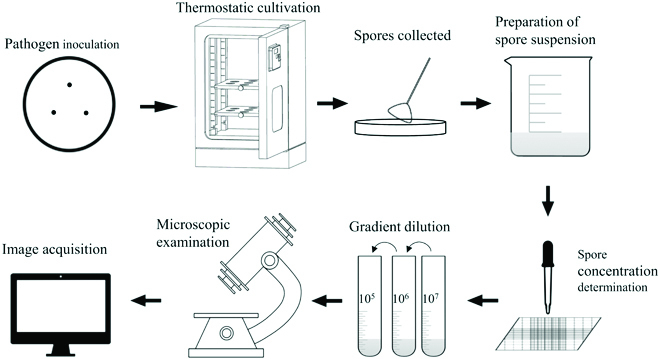
The gray mold spore extraction and microscopic image acquisition procedure.

**Fig. 2.**
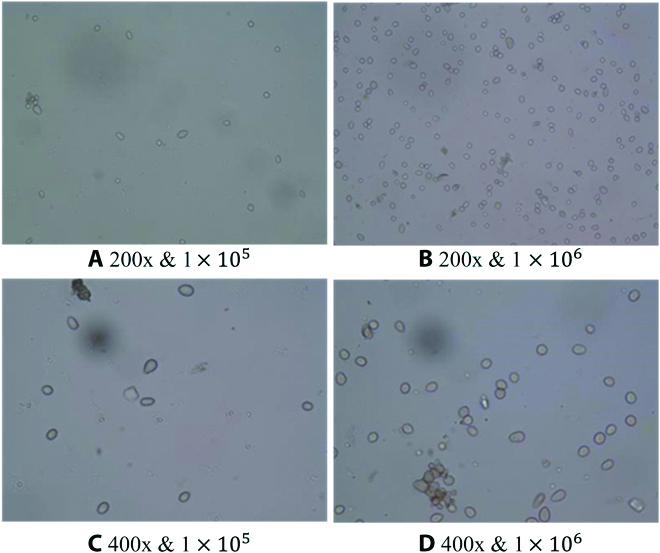
Examples of the microscopic images of *Botrytis cinerea* spore samples. (A) to (D) respectively represent samples collected under different concentrations and magnification conditions.

The microscopic images collection instrument is an Olympus fluorescence microscope BX51, magnifying 200× and 400×. 20μL of *Botrytis cinerea* suspension with 1 × 10^4^, 1 × 10^5^, and 1 × 10^6^ spores/ml were absorbed, respectively, dropped onto slides, and observed 12 visual fields on each slide. We finally obtained microscopic images with a resolution of 4,608 × 3,456. The procedure of gray mold extraction and image acquisition is shown in Fig. 1. A total of 886 spore samples were collected, of which 453 were magnified 200 times and 433 were magnified 400 times and saved in “.jpg” format. The examples of microscopic images of 200× and 400× are shown in Fig. 2.

#### Images annotation and dataset production

To obtain accurate detection data, the minimum external rectangle of the spore is used as the ground truth to manually label the image to reduce background information interference in the actual box. We labeled the spores of gray mold as *Botrytis cinerea* category. The background is not marked. This process annotates the dataset using LabelImg (https://github.com/tzutalin/labelImg). Finally, an XML file with the target type and coordinate information is saved.

**Fig. 3.**
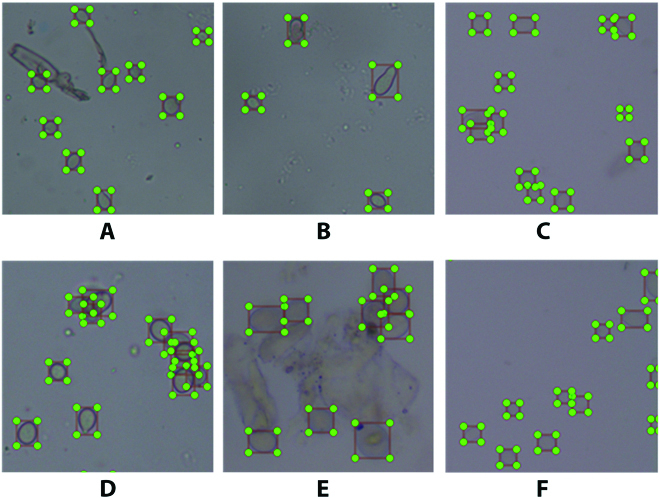
Some example of spore images, with green boxes representing the spores to be predicted. (A) to (F) indicate sample labeling of gray mold spores in various difficult situations, respectively.

Figure 3 illustrates the dataset. The standard gray mold spores is oval and clustered at the tip of the conidiophore. Influenced by light, spore extraction operations, and microscopy, the task of detecting gray mold spores has the following enormous challenges:

1. In addition to gray mold spores, the images also contain impurities, air bubbles, and other disturbing factors, as shown in Fig. 3A.

2. The spore morphology and size vary in *Botrytis cinerea* suspension, as shown in Fig. 3, B and C, because the spores are in different developmental stages.

3. In suspensions with higher concentrations, the spores show severe adhesions and occlusion. They are sometimes difficult to find even by humans, as shown in Fig. 3D.

4. When the camera focuses on some spores, other spores located at the edge of the image or away may be out of focus, making feature extraction very difficult, as shown in Fig. 3E.

5. When the spores are located at the edge of the image, the shape may be incomplete, as shown in Fig. 3F.

These cases add to the richness of the images in the dataset, making the algorithm development more challenging and practical. A total of 886 spore image samples were randomly divided into a training set (80%), validation set (10%), and test set (10%). Details of collected data are shown in Table 1. The average number of spores per image at 200× and 400× magnification was 53 and 17, respectively. The maximum number of spores per image at 200× magnification was 242, as in Fig. 2B. In deep learning training, fewer samples often lead to overfitting, so the sample images need to be augmented [29]. In this paper, we mainly use Mosaic, flipping up and down and left and right, to increase the number of spore images by online expansion.

**Table 1.** Number of images and spore instances in the dataset

Dataset		200×	400×	Total
Training set	Images	366	346	712
	Spore instances	18,630	5,630	24,260
Validation set	Images	44	44	88
	Spore instances	2,204	959	3,163
Test set	Images	43	43	86
	Spore instances	3,170	710	3,880

## The proposed MG-YOLO architecture for spore detection

**Fig. 4.**
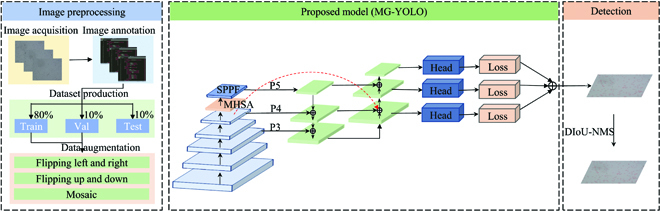
The overall architecture of proposed spore detection.

This section introduces the proposed MG-YOLO for gray mold spore detection. Figure 4 illustrates the overall architecture of the proposed detection for the gray mold spores, which mainly consists of 3 parts: image preprocessing, proposed model, and detection. In the preprocessing stage, the spore images were expanded using image enhancement methods. After preprocessing, the model (MG-YOLO) is constructed and the network weights are learned using the training and validation sets. The DIoU-NMS algorithm removes the redundant boxes at the detection end to produce the final detection results.

**Fig. 5.**
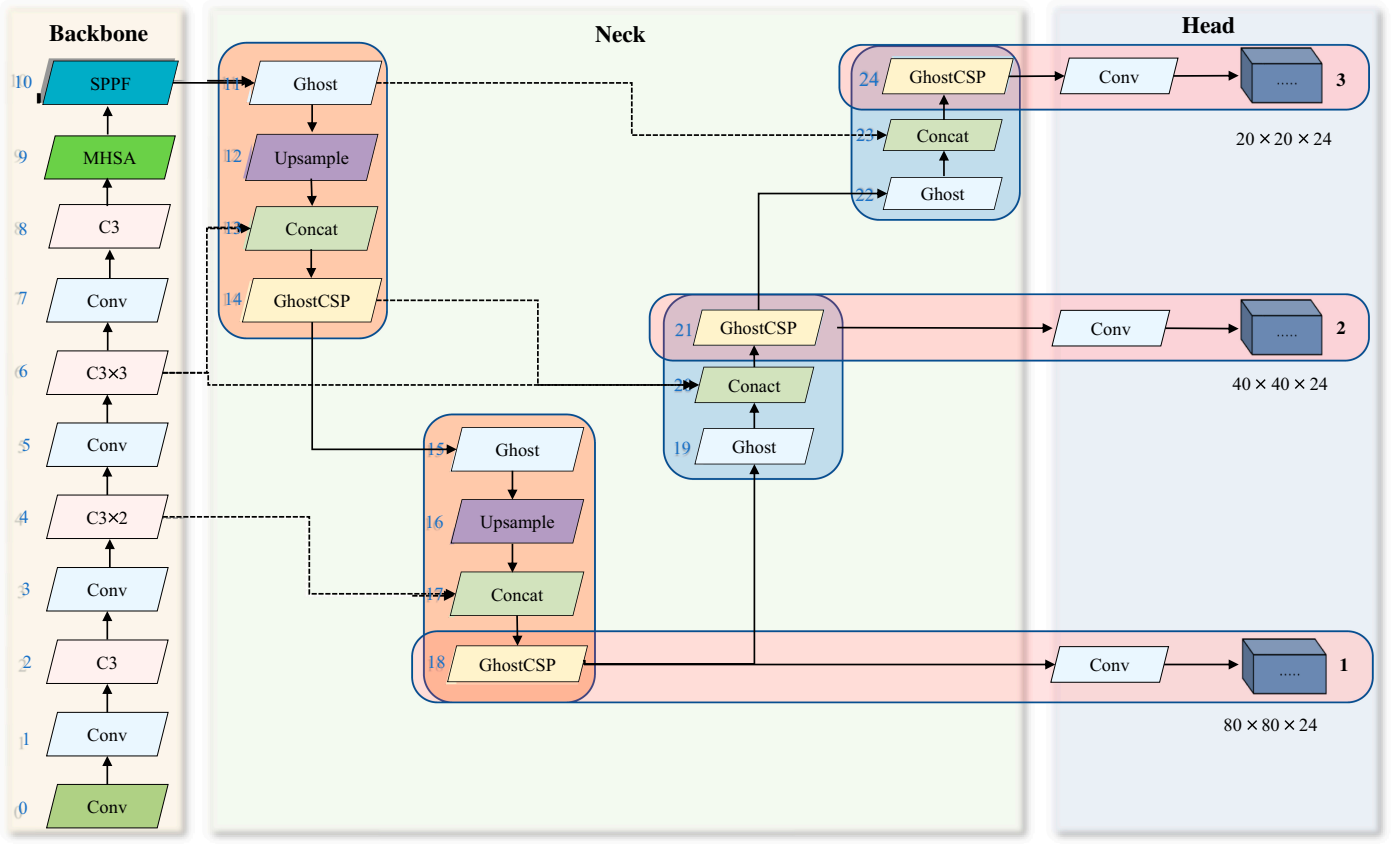
MG-YOLO architecture diagram.

In order to improve the accuracy and ensure the speed of detection of gray mold spores, based on the analysis of the dataset features and YOLOv5 network, we determined that the main optimization directions were feature extraction, feature fusion, and lightweight. Specifically, MHSA was added to the backbone to obtain global information. BiFPN was used to improve the connection of the neck for feature fusion. A lightweight model **G**host was used to optimize the Neck part to reduce the network complexity. Ultimately, the proposed **MG-YOLO** is shown in Fig. 5. In the next section, we describe the modules used in optimizing YOLO.

### Multi-head self-attention

**Fig. 6.**
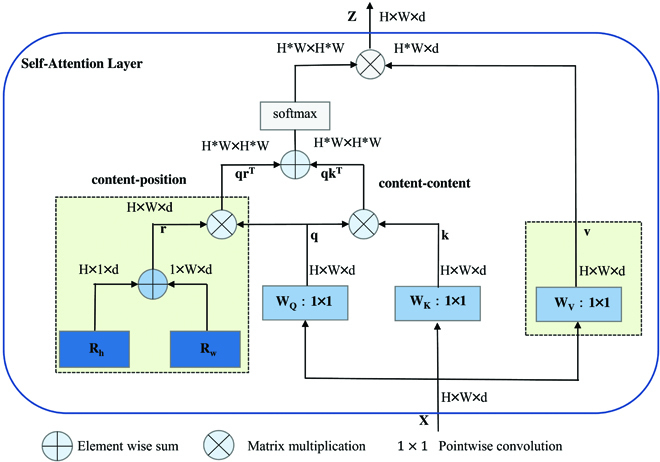
MHSA layer used in the MG-YOLO.

Vision tasks for target detection require modeling of remote dependencies. To be able to obtain global dependencies by aggregating local interactions, convolution-based network structures usually require stacking multiple layers. While this is effective, it is more convenient to build a model directly that can model global dependencies [30]. Inspired by the literature [31], we introduced MHSA into the backbone of YOLOv5 for capturing global information. MHSA is shown in Fig. 6. MHSA can process and aggregate the information contained in the convolutional captured feature maps using global self-attention.

This study aims to optimize the model's ability to extract spore features of gray mold using the self-attention mechanism. However, the computational complexity of self-attention is directly related to the length of the input features, so in this paper, self-attention is used only in the lowest resolution feature maps. Using convolution for spatial down sampling and focusing the self-attention on smaller resolutions can effectively handle large images.

### Ghost for lightening model

**Fig. 7.**
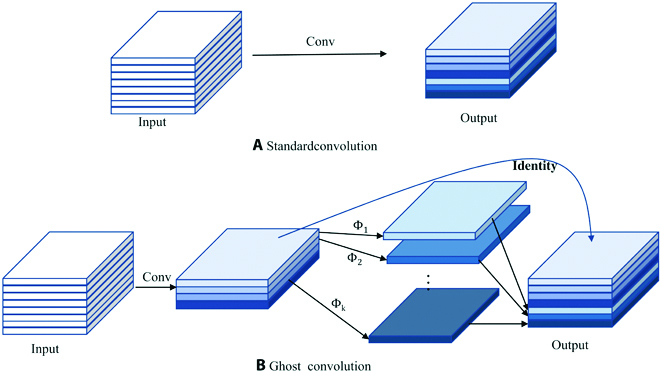
The Ghost convolution. (A) and (B) denote the standard convolution operation and Ghost convolution operation, respectively.

GhostNet proposes an innovative module Ghost, which generates more feature maps with fewer parameters and computations [32]. Ghost first obtains a part of the feature map by a standard convolution (Eq. 1). Then the acquired feature map is subjected to linear operations (Eq. 2) to generate more feature maps. The new feature maps are called ghost of the previous feature maps and are used to eliminate redundant features. Finally, the 2 feature maps are stitched together in the specified dimension. Ghost can reduce the cost of general convolutional computation while maintaining accurate performance [33]. Ghost as shown in Fig. 7.

$$Y^{\prime }=X∙f^{\prime }$$ (1)

$$Y_{\mathrm{ij}}=\Phi_{\mathrm{ij}}∙Y_{i}^{\prime },i\in \left[1,m\right],j\in \left[1,s\right]$$ (2)

**Fig. 8.**
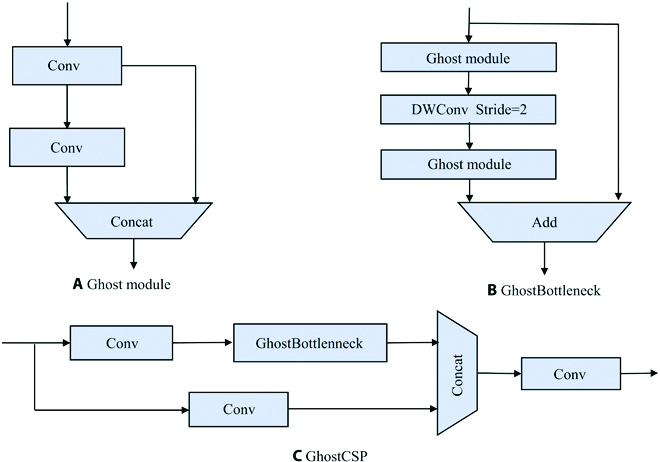
The structure of Ghost. (A) denotes the Ghost module, and (B) and (C) denote the GhostBottleneck and GhostCSP modules constructed in this paper, respectively.

Inspired by the Ghost module, this study has designed the GhostBottleneck and GhostCSP modules as shown in Fig. 8. The improved neck uses Ghost module and GhostCSP module to replace the regular convolutional layer and C3, respectively, which improves the detection speed.

### Weighted BiFPN for enhancing feature fusion

**Fig. 9.**
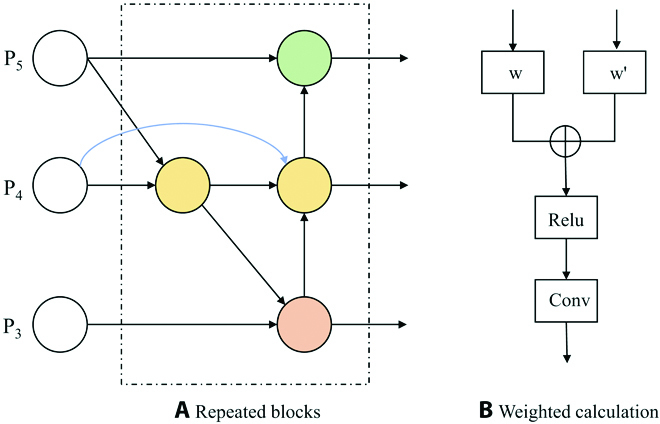
The structure of BiFPN.

An effective feature fusion structure is illustrated in Fig. 9A: the weighted BiFPN [34]. It has 2 major features: cross-scale connection and weighted feature fusion. The cross-scale connection can fuse deeper feature information from more scales. The output comes with additional weighted features to indicate the importance of different features, as shown in Fig. 9B.

YOLOv5 uses PANet to fuse features from top-down path and bottom-up path in order. The direct summation approach in fusion ignores the essential cases of features and increases the computational effort. We use BiFPN to improve PANet by removing the nodes of single input edges and adding additional edges to fuse higher-level feature. Information flow and feature aggregation are enhanced without adding extra cost; meanwhile, the model learns important features by weighting them. In this study, feature weights are calculated using fast normalized fusion with the following equation:

$$O=\sum_{i}\frac{\omega_{i}}{\varepsilon +\sum_{j}\omega_{j}}∙I_{i}$$ (3)

where, *ω_i_* ≥ 0, *I_i_* and *ω_i_* denote the feature values of the *ith* input and their weights, respectively. *ε* is taken as 0.0001 to prevent the formula from not working.

### Experimental setup

#### Experimental platform.

All experiments were conducted in the Ubuntu 18.04.5 environment (processor: 64 Intel(R) Xeon(R) Gold 6226R CPU @ 2.90GHz; memory: 251G; graphics card: Tesla V100-PCI-E-32GB*2). Based on Python 3.7 and the PyTorch 0.11.2 framework, we implement all the experiments in the paper.

#### Training settings.

In the process of training, the initial value of the learning rate was 0.01, and we used the cosine annealing strategy to reduce the learning rate. The stochastic gradient descent optimizer was adopted. We set 300 epochs, weight decay to 0.0005, learning rate momentum to 0.9, and batch size to 8. To prevent the training process of the model from being affected by overfitting caused by a large learning rate, the warmup learning rate was used, and the warmup epoch was set to 3. If no improvement is observed in the last 100 epochs, training was stopped early to save computational resources. In this paper, we used DIoU_NMS as the detection post-processing based on CIoU_Loss to improve the detection accuracy of overlapping targets effectively [35]. The confidence threshold was 0.25, and the IoU threshold was 0.45 during testing. The open source code can be available on the GitHub (https://github.com/D715-ky/fungal-spore-detection).

#### Evaluate metrics.

In this study, we calculated the AP [36] for 1 category, spores, to assess the effectiveness of the model. Model size and number of parameters are also a metric to characterize the model. Image detection time and FPS (Frames Per Second) are also used to characterize the speed of the target detection algorithm.

## Results and Discussion

In this section, we designed 3 experiments to test the detection performance of gray mold spores. In the first experiment, to select a baseline model for the accuracy and time balance of the gray mold detection, we validated and compared 5 models of the YOLOv5 series. In the second experiment to evaluate the validity and feasibility of the proposed model, we performed an ablation experiment to validate the performance of different sections. To study the detection performance of MG-YOLO, the performance was compared with state-of-the-art in the third experiment. Finally, to observe the MG-YOLO model's detection effect, the detection results were visualized in different data cases.

### Detection results of YOLO

The YOLOv5 series models were designed with 5 models based on model depth multiple and layer channel multiple, namely YOLOv5n, YOLOv5s, YOLOv5m, YOLOv5l, and YOLOv5x. The 5 models were trained using pretrained weights, and comparing the results can help us to choose the baseline model for gray mold spore detection.

Table 2 clearly shows that with the same hyperparameter settings, the detection accuracy of gray mold spores grows gradually with the model depth and width increase. At the same time, the model size also multiplies. YOLOv5n has the most miniature model with the lowest accuracy of 0.895. YOLOv5x achieves the highest AP of 0.981, but its model is as high as 173.1 M. From the comparison between YOLOv5n and YOLOv5s, the AP increases by 2%, the required model size increases by 10.5 M, and the network parameters increase by 5.25 × 10^6^. Compared to YOLOv5x, which has the highest accuracy, YOLOv5s reduces the model storage space by 158.7 M at the cost of losing 0.066 AP values. After balancing the model size and detection accuracy, YOLOv5s was selected as the baseline model for gray mold spore detection.

**Table 2.** Detection results of YOLOv5 series models

Method	AP	Model size (M)	Parameter (×10^6^)
YOLOv5n	0.895	3.9	1.76
YOLOv5s	0.915	14.4	7.01
YOLOv5m	0.956	42.2	20.9
YOLOv5l	0.976	92.9	46.11
YOLOv5x	0.981	173.1	86.17

### Ablation experiment

The proposed model contributes 3 elements, including MHSA, Ghost-optimized neck (GNeck), and weighted BiFPN. To analyze the contribution of each component, we performed an ablation study based on the experimental results in the previous section, adding each of the 3 parts to the baseline model.

**Fig. 10.**
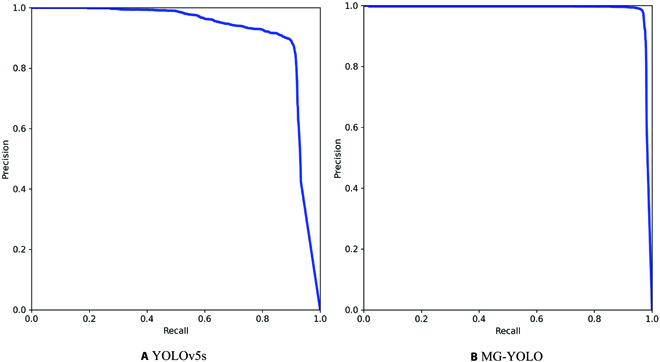
PR curves of baseline model (YOLOv5s) and proposed model (MG-YOLO).

The results obtained are shown in Table 3. We first added MHSA to the backbone (YOLOv5s-a), and the AP improved by 6.2%. The model size increased by 0.8 M. This is because MHSA uses global self-attention to the information in the feature map processing and aggregation, significantly improving the model's ability to process the features of gray mold spores. When both MHSA and GNeck were added to YOLOv5s (YOLOv5s-b), the AP increased by 5.6%, and the model size decreased by 19 M. Compared with YOLOv5-a, it was able to reduce the model size and parameter by 2.7 M and 1.41 × 10^6^, respectively, with a loss of only 0.006 AP. This is due to Ghost's ability to maintain accurate performance while reducing the general convolutional computation cost. As shown in the last row of Table 3, the AP was further improved by adding MHSA, GNeck, and BiFPN most simultaneously in YOLOv5s, and the final AP reached 0.983. BiFPN enhanced the feature representation and facilitated the detection of gray mold spores. Compared with YOLOv5s, MG-YOLO improved the accuracy by 6.8% and reduced the model size by 1.8M. MG-YOLO balanced the model parameters and accuracy, indicating that the proposed improvements are effective. Figure 10 shows the PR curves of the original YOLOv5 and the proposed MG-YOLO. The area between the curve and the X and Y axes represents AP, and the larger the area, the higher the detection accuracy. This also means that our proposed MG-YOLO outperforms the baseline detector.

**Table 3.** Evaluation results of ablation experiments

Method	MHSA	GNeck	BiFPN	AP	Model size (M)	Parameters (×10^6^)
YOLOv5s	×	×	×	0.915	14.4	7.01
YOLOv5s-a	√	×	×	0.977	15.2	7.41
YOLOv5s-b	√	√	×	0.971	12.5	6.00
MG-YOLO	√	√	√	0.983	12.6	6.10

### Comparison with the state-of-the-art

To fully validate the performance of the proposed method, we compare the proposed MG-YOLO with 6 common 1-stage and 2-stage detection models. The Faster RCNN [17], RetinaNet [20], YOLOv3 [37], Dynamic RCNN [38], YOLOv5, and YOLOX [39] are included. All the parameters of the comparison methods are consistent with the original settings without any adjustment, which helps to ensure the credibility of the experimental results [24]. The quantitative results of the state-of-the-art detection algorithm in the test set are shown in Table 4.

**Table 4.** Comparisons with state-of-the-art detection methods

Method	Backbone	AP	Parameters (×10^6^)	FPS (img/s)	Detect time (s)
Faster RCNN	Resnet50	0.872	41.34	22.2	0.045
Dynamic RCNN	Resnet50	0.86	41.35	60.3	0.045
RetinaNet	Resnet50	0.829	36.33	23.4	0.043
YOLOv3	Darknet53	0.871	61.52	22.1	0.017
YOLOv5	CSPDarknet53	0.915	7.01	138.9	0.007
YOLOX	Darknet53	0.929	8.94	89.3	0.011
MG-YOLO	CSPDarknet53	0.983	6.10	116.3	0.009

This study aimed to enhance the YOLOv5 model to identify gray mold spores using a self-built dataset accurately. In Table 4, MG-YOLO achieved an AP of 0.983, detecting an image in only 0.009 s with an FPS of 116.3. YOLOv5 and YOLOX are high-performance models proposed in recent years with relatively good detection accuracy (AP > 0.9), with APs of 0.915 and 0.929, respectively. The AP of MG-YOLO is 6.8% and 5.4% higher than theirs, respectively. The proposed MG-YOLO has significantly better detection accuracy and speed than Faster RCNN, Dynamic RCNN, RetinaNet, and YOLOv3. The accuracy of Faster RCNN, Dynamic RCNN, RetinaNet, and YOLOv3 in detecting gray mold spores was similar with APs of 0.872, 0.86, 0.829, and 0.871, respectively, and the AP of the proposed model is at least 0.111 higher than theirs. Accurate spore detection provides statistical information about the spores, such as their numbers. In practical application, the detection model can be a good substitute for manually accurately acquiring the number of spores, saving labor and time costs.

Faster RCNN, Dynamic RCNN, and RetinaNet have similar speeds, which take about 0.043 s to detect an image. YOLOv3, YOLOv5, and YOLOX can detect images more quickly. However, their parameter sizes are larger than those of the proposed model, and their APs are not as good as the proposed model. Therefore, comparing the AP, parameter size, and detection time, the proposed MG-YOLO model maintains high accuracy while maintaining the real-time performance of the detection task. This is because the MHSA improves the model's expressiveness and increases the model's ability to capture different local information. The weighted BiFPN not only fuses multiscale feature information but also learns important features. Meanwhile, Ghost optimized neck, which can reduce the number of parameters of the model and improve the detection speed.

### Visualization of detection results

**Fig. 11.**
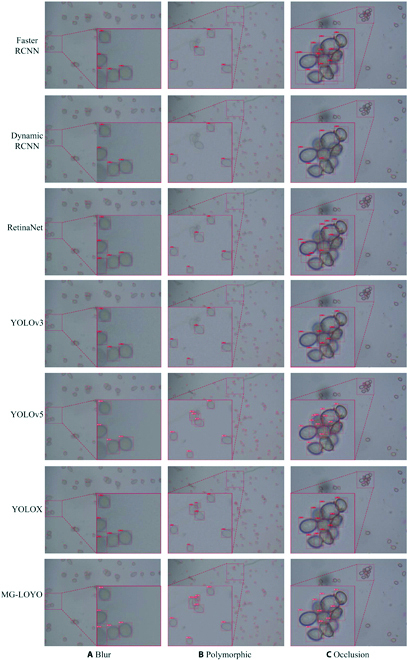
Results of gray mold spore detection in different cases (A) to (C) indicate the gray mold spores in the blur case, the polymorphic case, and the occlusion case, respectively.

**Fig. 12.**
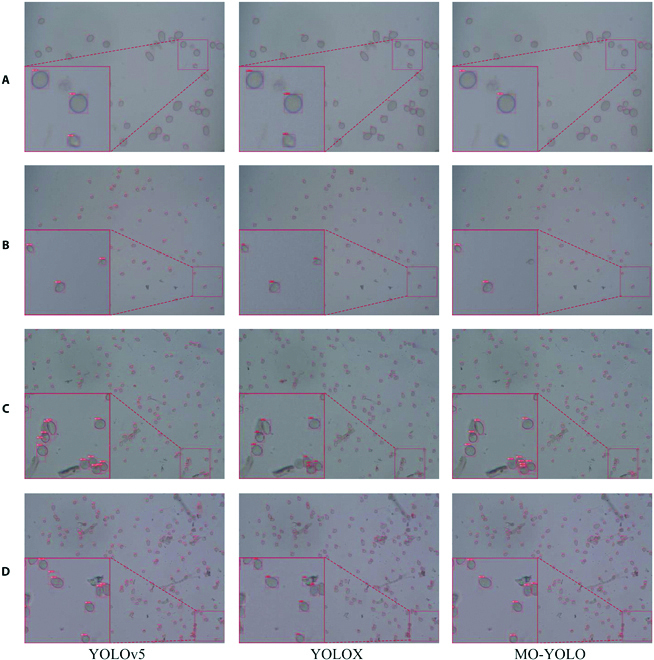
Visualization of model detection results at different densities. (A) to (D) indicate images with spore counts less than 50, between 50 and 100, between 100 and 150, and greater than 150, respectively.

We visualize the results of the spore detection as shown in Figs. 11 and 12. Figure 11 shows the detection plots of the proposed model and the comparison model in the complex case of gray mold spore images. Figure 11 shows that Faster RCNN, Dynamic RCNN, RetinaNet, and YOLOv3 have different degrees of missed detection due to blurring, small target, and occlusion. The spores were more likely to be missed in small targets, large numbers, or edge positions (Fig. 11A and B). YOLOv5, YOLOX, and MG-YOLO performed better in detecting gray mold spores. However, RetinaNet, YOLOv5, and YOLOX had redundant boxes in the case of multiple spore adhesions and misjudged images of dense spores and spores with polymorphic (Fig. 11B). Figure 11C shows that the comparison models all have some degree of missed detection or redundancy for adherent spores.

Compared with YOLOv5, the detection visualization diagram shows that increasing AP by 6.8% can significantly reduce missed detection and invalid detection boxes (Fig. 11). The MG-YOLO can effectively identify both large and small targets, is not affected by blur, multimorphology, or background noise, and has an advantage in detecting spores obscured by adhesion. This is crucial for spore information statistics, which can accurately assess the degree of resistance and activity. This proves that the incorporation of MHSA can enhance the ability of the model to extract global information and improve the detection accuracy of the model for small targets and polymorphs. BiFPN motivates the model to effectively process shallow features and deep semantic information to improve the detection ability of the model for occlusion targets.

The detection of pathogen spores in the literature [11–13] is limited to small density images at high magnification. The rapid and accurate detection of small pathogen spores at high densities has not been addressed. To verify the effectiveness of the proposed MG-YOLO for detecting small pathogen spores at high densities, we further present the visualization results of the proposed model at different densities. Table 4 and Fig. 11 show that YOLOv5, YOLOX, and MG-YOLO perform better in the gray mold detection task. Therefore, Fig. 12 only shows the detection results of these 3 models. Depending on the number of spores in each image, we classified the images into 4 densities, which are (a) spore count less than 50, (b) spore count between 50 and 100, (c) spore count between 100 and 150, and (d) spore count greater than 150 (Fig. 12). As seen in Fig. 12A, all 3 models could accurately detect gray mold spores when the spore density was small. As the density increases, YOLOv5 starts to show errors and misses, and the errors become more and more frequent. This is because as the spore density increases, the impurities in the images also increase, while the adhesion of spores becomes more and more common. YOLOX starts to show different degrees of errors in densities c and d. Moreover, the proposed MG-YOLO can better cope with high density (Fig. 12C and D). In general, the MG-YOLO model is better at detecting different densities. Therefore, the method described in this paper has strong robustness and can be adapted to detect gray mold spores in different scenarios.

Although the proposed MG-YOLO outperformed the comparison model on the self-constructed gray mold dataset, the generalization ability of the model needs further validation, such as different classes of fungal spores and different developmental periods of fungal spores of the same species. Since there are many types of fungal spores and small differences among some fungal spores, the algorithm performance may be degraded by applying the proposed model directly to other classes of spores. In this case, the generalization of the model can be further improved by methods such as transfer learning and knowledge distillation.

## Conclusion

To explore the capability of the object detection algorithm in pathogen spore detection and to solve the blurring, multiform, and occlusion problems in the actual scenarios of spore detection, a spore detection algorithm named MG-YOLO is proposed in this paper. The model used MHSA to enhance the processing of global information of spores of gray mold and weighted BiFPN to enhance the feature extraction of spores of different scales. The model used Ghost lightweight network to optimize the neck part, retaining high accuracy while reducing the number of parameters of the whole network. Extensive experimental results show that proposed model achieves an AP value of 0.983, a 6.8% improvement, which is better than other comparative models. At the same time, the number of parameters is minimal, only 6.1 × 10^6^, and the FPS reaches 116.3, which is more practical in detecting gray mold spores in multiple scenarios and densities. The improved model provides a theoretical basis for pathogen spore detection and statistical information, vital for early disease diagnosis and breeding resistant varieties.

In the next step of our research, we will further collect more spore images to increase the variety of disease the model can detect. Meanwhile, the model will be further migrated and implemented to enhance the algorithm's utility, reduce personnel costs, and improve economic advantage. Based on this study, we will explore the dynamic law of pathogen spores infecting crops.

## Data Availability

The data used in this study are available from the corresponding author upon reasonable request. The open source code can be available on the GitHub (https://github.com/D715-ky/fungal-spore-detection).
